# Rhamnolipid the Glycolipid Biosurfactant: Emerging trends and promising strategies in the field of biotechnology and biomedicine

**DOI:** 10.1186/s12934-020-01497-9

**Published:** 2021-01-04

**Authors:** Priyanka Thakur, Neeraj K. Saini, Vijay Kumar Thakur, Vijai Kumar Gupta, Reena V. Saini, Adesh K. Saini

**Affiliations:** 1 School of Biological and Environmental Sciences, Faculty of Sciences, Shoolini University, Solan, HP 173229 India; 2 School of Biotechnology, Jawaharlal Nehru University, New Delhi, 110 067 India; 3 Biorefining and Advanced Materials Research Center, Scotland’s Rural College (SRUC), Kings Buildings, West Mains Road, Edinburgh, EH9 3JG UK; 4 Centre for Safe and Improved Food, Scotland’s Rural College (SRUC), Kings Buildings, West Mains Road, Edinburgh, EH9 3JG UK; 5 Department of Biotechnology, MMEC, Maharishi Markandeshwar (Deemed to be University), Mullana, Haryana 133207 India; 6 Maharishi Markandeshwar University, Kumarhatti, Solan, Himachal Pradesh 173229 India

**Keywords:** Rhamnolipids, Antimicrobial agents, Antitumor agents, Biosurfactants, Immunomodulators

## Abstract

Rhamnolipids (RLs) are surface-active compounds and belong to the class of glycolipid biosurfactants, mainly produced from *Pseudomonas aeruginosa*. Due to their non-toxicity, high biodegradability, low surface tension and minimum inhibitory concentration values, they have gained attention in various sectors like food, healthcare, pharmaceutical and petrochemicals. The ecofriendly biological properties of rhamnolipids make them potent materials to be used in therapeutic applications. RLs are also known to induce apoptosis and thus, able to inhibit proliferation of cancer cells. RLs can also act as immunomodulators to regulate the humoral and cellular immune systems. Regarding their antimicrobial property, they lower the surface hydrophobicity, destruct the cytoplasmic membrane and lower the critical micelle concentration to kill the bacterial cells either alone or in combination with nisin possibly due to their role in modulating outer membrane protein. RLs are also involved in the synthesis of nanoparticles for in vivo drug delivery. In relation to economic benefits, the post-harvest decay of food can be decreased by RLs because they prevent the mycelium growth, spore germination of fungi and inhibit the emergence of biofilm formation on food. The present review focuses on the potential uses of RLs in cosmetic, pharmaceutical, food and health-care industries as the potent therapeutic agents.

## Background

Surfactants are group of amphipathic compounds having hydrophobic and hydrophilic moieties with the potential to lessen the surface and interfacial tensions in molecules [1, 2] which makes them very suitable for lubrication, emulsification, foaming, detergency and dispersing agents and thus, can be effectively used by food, petroleum, agriculture, pharmaceutical industries as well as in the environmental remediation [3, 4]. Initially, petrochemical based synthetic detergents were used in the industries, but they are toxic and non-biodegradable. But their negative effects on the environment made researchers to introduce biosurfactants which are surface-active compounds having low toxicity and high biodegradability. Biosurfactants can be synthesized either enzymatically or can be isolated from microorganisms [1–3, 5].

Biosurfactants harbour hydrophobic (saturated/unsaturated fatty acids) and hydrophilic (amino acids/peptides, anions/cations, di-/polysaccharides) moieties which are synthesized by fungi, bacteria, yeast. They decrease the surface and interfacial tensions in solid/liquid, liquid/liquid, liquid/gas phases [6–8]. Diverse properties of biosurfactants namely high biodegradability, low toxicity, low critical micelle concentration (cmc), low cost production, and tolerance to extreme conditions (high temperature and salinity, low and high pH) make them effective to be used over petro-chemical surfactants [4, 9–11]. Moreover, these biosurfactants also show antitumor and antimicrobial activity [12, 13]. Biosurfactants are classified based on the following criteria: (a) ionic charge, (b) molecular weight, (c) secretion type (adhesion to microbes, intra and extracellular) and (d) chemical structure [8]. Amongst all, the low molecular weight biosurfactants are mostly studied and further divided into glycolipids and lipopeptides group [3].

### Glycolipid biosurfactants

Glycolipids are the most worked out biosurfactants of low molecular weight and are synthesized from hydrocarbons, industrial wastes, frying and olive oil wastes [8]. Structural composition of glycolipid biosurfactants consists of hydrophilic moiety having carbohydrate compounds like glucose, mannose, galactose, trehalose, rhamnose, sophorose and hydrophobic moiety having long fatty acid chain [14, 15]. Glycolipids are shown to be very efficient against various bacteria, viruses, mycoplasma and fungus because of their potent role in destabilizing the biological membranes via the generation of ion channels and pores [8]. Glycolipids activate/inhibit enzyme for various biotechnological processes by modulating the enzyme activities. They are also used in cosmetics because of their moisturization ability as well as because of their anti-adhesive property which inhibit the bioadhesion of any bacteria [8].

Glycolipid biosurfactants are further subdivided into rhamnolipids, trehalose lipids, sophorolipids, mannosylerythritol lipids, cellobiose lipids, monoacylglycerol, diglycosyl diglycerides, lipomannosyl-mannitols, galactosyl-diglyceride, lipoarabinomannanes and lipomannans [3, 8]. In the recent past, rhamnolipids are extensively studied because of their lower range in both surface tension (28 to 30 mN/m) as well as critical micellar concentration (10 to 200 mg/L) and, high emulsifying indexes from 60–70% as well as their high production in very short duration [16].

#### Rhamnolipids (RLs)

**Fig. 1:**
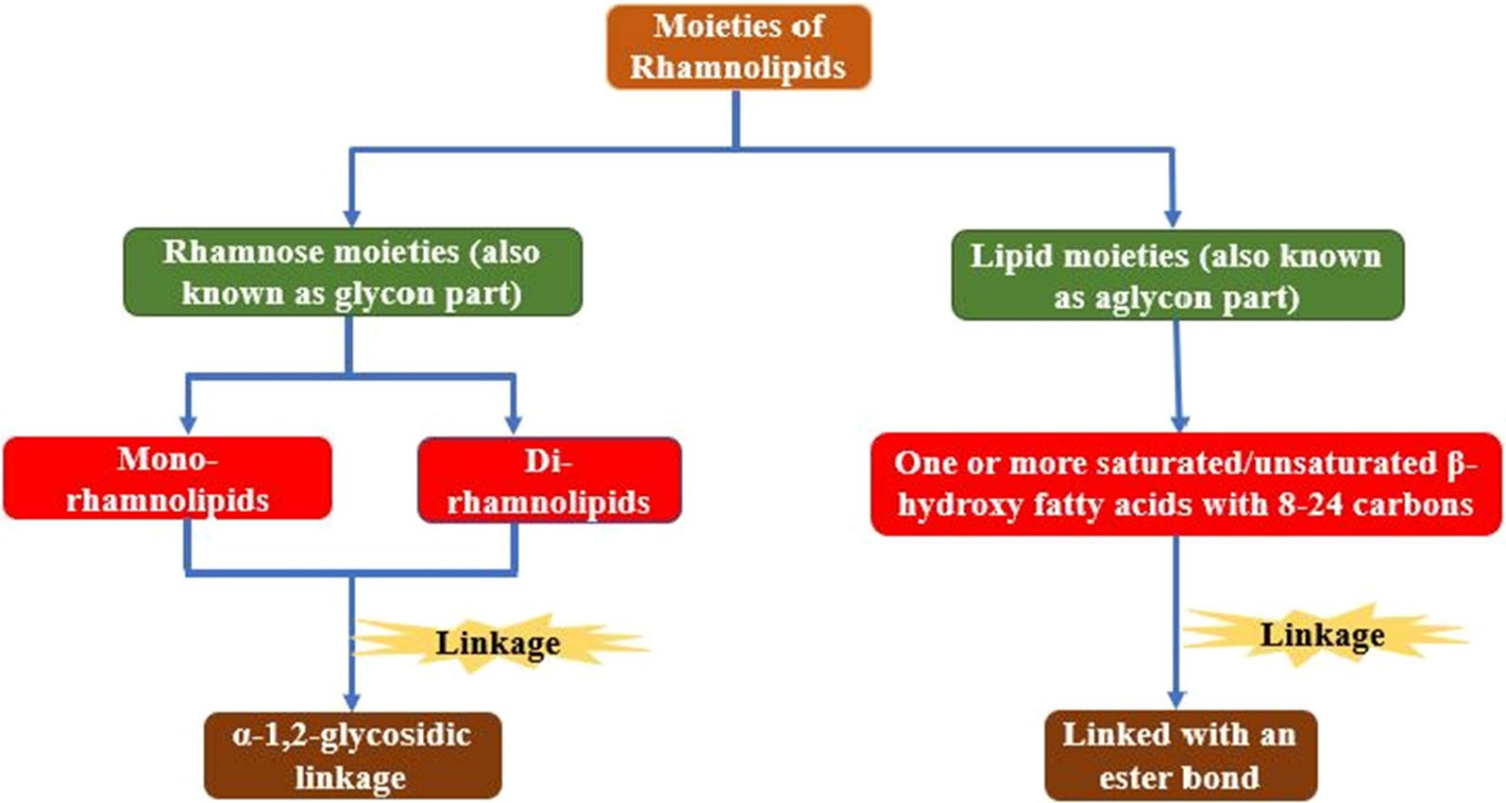
Flow chart showing rhamnolipid moieties

RLs are one of the most studied microbial amphipathic biosurfactants which was reported as “oily glycolipids” by Bergstrom et al. in 1946 [17, 18]. They have two moieties: Rhamnose (also known as glycon part) and lipid (also known as aglycon part) (Fig. 1) [4, 19]. Rhamnose moiety is hydrophilic in nature comprising of mono or di (L)-rhamnose molecules which are linked together through α-1,2-glycosidic linkage. The lipid moiety is hydrophobic in nature and comprises of one or more saturated/unsaturated β-hydroxy fatty acids chains of C_8−_C_24_ lengths, linked together with an ester bond (Fig. 1) [16, 19]. Both moieties are linked via glycosidic linkage [19]. By using varieties of sugar or hydrocarbons, different organisms can produce approximately sixty congeners or homologs of rhamnolipids [19–23]. Gram-negative bacterium *Pseudomonas aeruginosa* (*PA*) is the most predominant species which produces four familiar rhamnolipids, viz. 3-[3-(2-*O*-α-L-Rhamnopyranosyl-α-L-rhamnopyranosyloxy)decanoyloxy]decanoic acid (Rha2-C10-C10), 3-[(6-Deoxy-α-L-mannopyranosyl)oxy]decanoic acid (Rha-C10), 3-[3-(α-L-Rhamnopyranosyloxy)decanoyloxy]decanoic acid (Rha-C10-C10) and 3-[(2-*O*-α-L-Rhamnopyranosyl-α-L-rhamnopyranosyl)oxy] decanoic acid (Rha2-C10) [16, 22].

## Biosynthesis of rhamnolipids

**Fig. 2:**
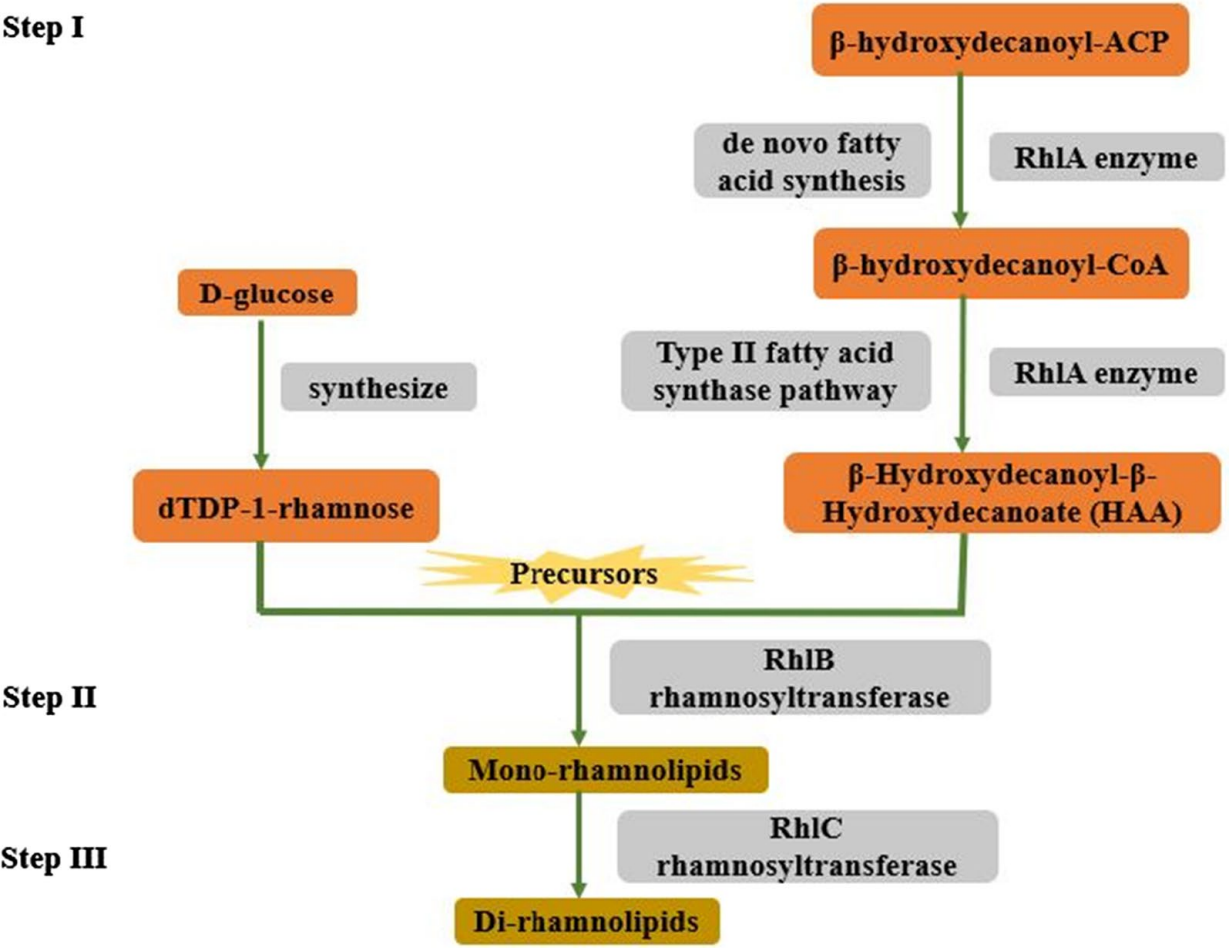
Steps for the biosynthesis of rhamnolipids

Different carbon sources can affect the supply of basic precursors for the biosynthesis of rhamnolipids and because of this reason, different *PA* strains produce variants of rhamnolipid [24]. A complex genetic network is required for RLs production including the *rhl* genes (*A*, *B* and *C*), quorum sensing and, three main steps which give rise to dTDP-l-rhamnose and variants of 3-(3-hydroxyalkanoyloxy) alkanoic acid (primarily β-Hydroxydecanoyl-β-Hydroxydecanoate; HAA) [4, 23, 25]. The three steps (as shown in Fig. 2) are: (a) RhlA enzyme transfers the β-hydroxydecanoyl present on the acyl carrier protein (ACP) to the coenzyme A (CoA) and forms β-hydroxydecanoyl-CoA intermediate in de novo fatty acid synthesis [25, 26]. RhlA directs the formation of β-Hydroxydecanoyl-β-Hydroxydecanoate (HAA) (a part of rhamnolipid) from the type II fatty acid synthase pathway [25, 27, 28] whereas d-glucose synthesized dTDP-1-rhamnose [29]. (b) RhlB rhamnosyltransferase involves the synthesis of mono-RL by using dTDP-1-rhamnose and 3-(3-hydroxyalkanoyloxy) alkanoic acid as precursors [30]. (c) RhlC rhamnosyltransferase directs the condensation of mono-RLs and dTDP-1-rhamnose to synthesize di-rhamnolipids [31].

Rhamnolipids production is transcripShreya delete from heretionally regulated through quorum sensing signals [32–34]. RhlI and LasI enzymes are responsible for the synthesis of autoinducer molecules; autoinducer molecules when outreach the thresshold concentration persuade the *rhl* genes expression by binding to their regulatory proteins LasI and RhlR [26, 35, 36].

## Applications of gycolipids biosurfactant: rhamnolipids

RLs have tremendous applications [37–40]. Rhamnolipids have the potential to reduce the surface tension between solid/liquid, liquid/liquid, liquid/gas phases which lead to their role in cosmetics, detergent and other washing agents producing industries [41, 42]. Rhamnolipids also have the potential to fold the outer membrane protein OmpA [43]. Beside these properties, they also have antimicrobial, anticancer, immunomodulation as well as they have ability to synthesize nanoparticles [44–47]. In this review paper, we have discussed different applications of rhamnolipids at one place which were not discussed previously.

### Rhamnolipids as antitumor agents

Cancer is a dreadful disease, and RLs have shown a very potent effect on human and animal cancer cells [12]. Di-rhamnolipids produced from *PA* B189 impeded the proliferation of insect cell line C6/36 and breast cancer cell line MCF-7. RLs obtained from B1898 could also heal the wounds, recognize the cytoskeleton of phagocytic/non-phagocytic cells and can change their morphology [12, 48]. These properties of RLs, were further tested on various cells derived from tumors of different origin [12].

#### Antitumor activity against HL-60, SKW-3, B-173 and JMSU-1 human cancer cell lines

Christova et al., examined RL-1, a mono-rhamnolipid and, RL-2, a di-rhamnolipid BN10 strain for their antitumor effects on HL-60, SKW-3, BV-173, JMSU-1 human cancer cell lines [12] and the cytotoxicity assay revealed that RL-1 is more effective than RL-2 as shown in Table 1. RL-1 had higher toxic effect on BV-173 and SKW-3 cancer cell lines, but had lower toxicity effect on HL-60 and JMSU-1 cancer cell lines. Moreover, RL-2 utilized higher concentrations to show their cytotoxic effect as compared to RL-1. RL-1 induced the alterations in the morphology of leukemic cells like blebbing of the plasma membrane, chromatin condensation, the occurrence of apoptotic bodies and nuclear fragmentation leading to the apoptosis of BV-173 cells [12]. Interestingly, they found that higher concentrations of RL-1 lead to the overexpression of *c-myc* and *Bcl-2* genes of BV-173, resulting in rapid multiplication and anti-apoptosis of BV-173 cells [12]. These properties make RL-1 more potent to be used as antitumor agent in the field of biomedicine.

**Table 1:** Half-maximal inhibitory concentration (IC_50_) values of anticancer activity of RL-1 and RL-2 [12]

Rhamnolipid type	Cancer cell lines	IC_50_ values
RL-1	BV-173	IC_50_ = 50 µM
	SKW-3	IC_50_ = 54 µM
	HL-60	IC_50_ = 67 µM
	JMSU-1	IC_50_ = 60 µM
RL-2	BV-173	IC_50_ = 82 µM
	SKW-3	IC_50_ = 108 µM
	HL-60	IC_50_ = 77 µM
	JMSU1	IC_50_ = 140 µM

#### Antitumor activity against human breast cancer cells MCF-7

**Fig. 3:**
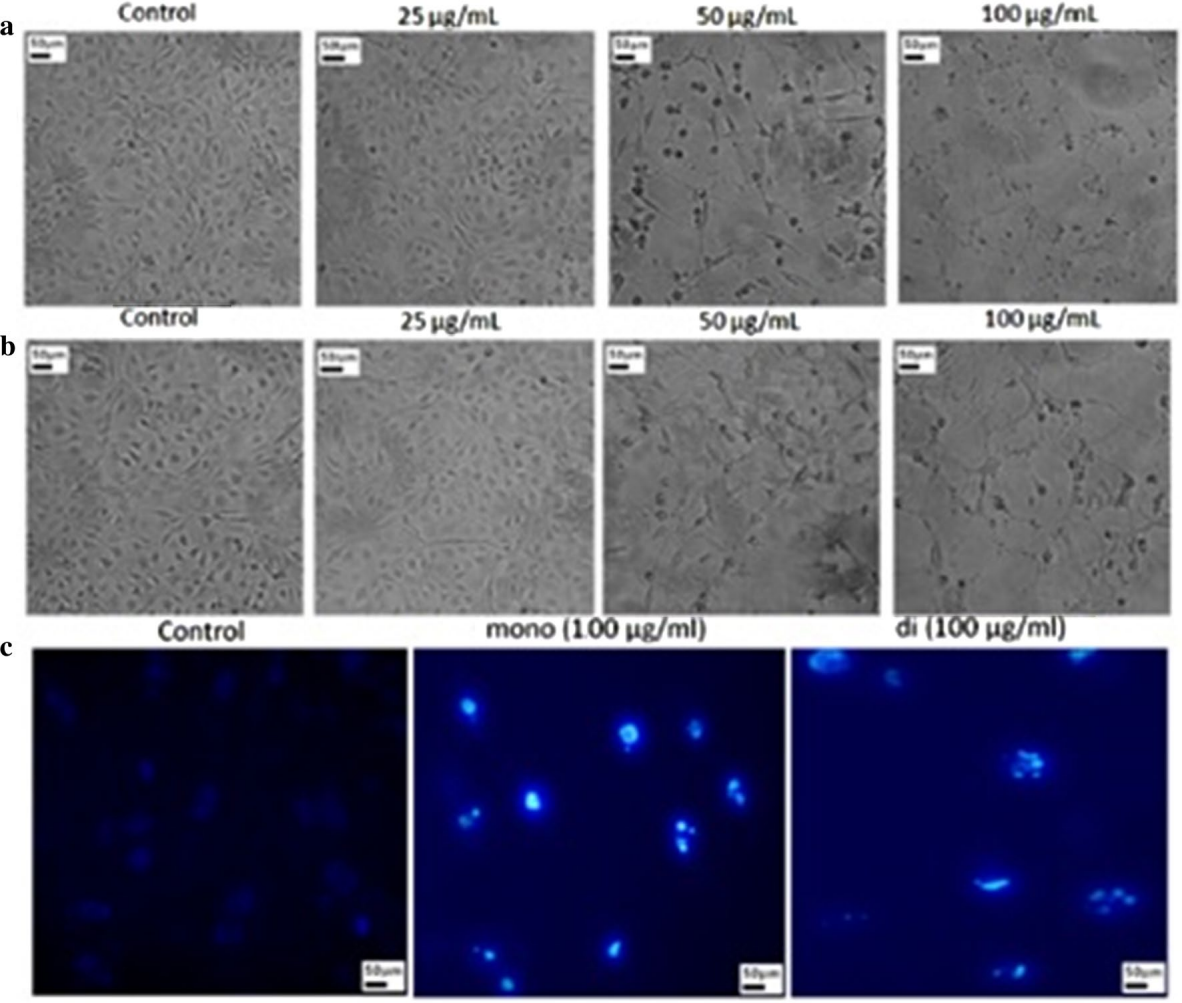
Phase contrast microscopy of MCF-7 cells showing morphological changes in shapes after treatment with **a** mono-rhamnolipids and **b** di-rhamnolipids for 48 h, **c** observation of morphological changes in rhamnolipids treated MFC-7 cells at 100 µg/mL concentration for 48 h [49]. "(Reprinted (Colloids and Surfaces B: Biointerfaces) with the permission from Elsevier (License number 4,918,290,880,371)”

Rahimi et al., in their study, isolated RL1 and RL2 from MR01 strain of *PA* and tested their cytotoxicity against MCF-7 cells [49]. They found that although both RLs inhibited the proliferation of MCF-7 cells but RL1 was more effective in killing the cancer cells [49]. They found that the pronounced effect on the cell viability was through their interaction with cell membrane. Results of phase-contrast microscopy revealed the changes in the morphology of MCF-7 cells after their treatment with mono- and di-rhamnolipids at 25, 50, 100 µg/mL concentrations for 48 h [49, 50]. Rhamnolipids treated MCF-7 cells have round and shrunken shapes whereas control MCF-7 cells have polygonal and cuboidal shapes as shown in Fig. 3a, b [49, 50]. Rhamnolipids treated MCF-7 cells lost their contact with adjacent cells resulted in their detachment from the surfaces leading to their flotation [49]. These morphological changes are signs of apoptotic cells which suggested that rhamnolipids have the potential to inhibit the MCF-7 proliferation [49, 51]; these apoptotic morphological changes were further observed through fluorescent microscopy using Hoechst staining [49]. Rhamnolipids treated cells exhibited strong nuclear fragmentation against the control cells, as shown in Fig. 3c indicating the induction of apoptosis. They also found the overexpression of p53 gene, which is a strong tumor suppressor gene, indicating a promising role of RLs in inducing the cell cycle control in cancer cells.

Thanomsub et al., also examined the inhibition efficiency of di-rhamnolipid “a” and di-rhamnolipid “b” produced from B189 strain of *PA* against MFC-7 cells [48]. Their results described that the growth of MCF-7 cells was efficiently inhibited by di-rhamnolipid “a” but not by di-rhamnolipid “b” [48]. Moreover, rhamnolipids produced from *PA* M14808, having Rha-Rha–C_10_–C_10_ (as a major part), also had inhibitory effect against MFC-7 cells [52].

A study done by Lotfabad et al., reported that rhamnolipid-type biosurfactant (MR01 biosurfactant) synthesized from *PA* MR01 inhibited the growth of HeLa cancer cells at 5 µg/mL because of their cytotoxic activities [44]. Based on all these studies, we can conclude that rhamnolipids could be used as potential therapeutic antitumor agents.

### Rhamnolipids as immunomodulators

Based on the divergent effects of rhamnolipids, several recent studies focused on the role of rhamnolipids as immunomodulators. These biosurfactants have profound effects on modulating the humoral and cellular immune systems, rhamnolipids can activate the immune cells resulting in the secretion of pro-inflammatory cytokines [46, 53], for example, rhamnolipids from *Burkholderia plantarii* could stimulate human mononuclear cells to secrete tumor necrosis factor α [46].

Rhamnolipids when preincubated with monocytes can increase oxidative responses of these cells along with the opsonization of zymosan by several folds [54]. This biosurfactant shown to be involved in inducing histamine release by mast cells [55] or inducing the production of serotonin and 12-hydroxyeicosatetraenoic acid by platelets [56]. During *in-vitro* infection of mouse peritoneal macrophages which were preincubated with rhamnolipids, significant reduction was seen in the phagocytosis of the bacteria [57]. Authors also examined the effect of rhamnolipids on the internalization of zymosan particles when injected intratracheally into the rat lungs at the physiological concentration. Their results indicated the inhibitory effect of RLs on alveolar macrophages in terms of the internalization of zymosan particles [57]. Several groups have shown that the rhamnolipids can cause the lysis and necrosis of macrophages and polymorphonuclear leukocytes respectively [58, 59]. Moreover, study by Dossel et al., revealed that rhamnolipids, inhibit the interaction of diacylglycerol (DAG) with protein kinase-C leading to decreased formation of human beta defensin-2 [60].

### Antifungal activity of rhamnolipids

Fungal pathogens cause the post-harvest decay of fruits and vegetables which affect the World’s food production by 5–10% [61]. Chemical fungicides are used to cure the post-harvest decay, but these have some consequences like toxicity, ground water pollution, threats to human health, prolonged degradation interval and development of fungicide-resistant strains [62–65]. Now-a-days, rhamnolipids are preferred over chemicals fungicides because of their antifungal activity against fungi like *Alternaria alternata, Mucor circinelloides* and *Verticillium dahlia*, inhibition of fungicides-resistance against chemical pesticides and stimulation of immunity in plants against plant pathogens [66–69].

#### Antifungal activity against *Alternaria alternata* fungus

**Fig. 4:**
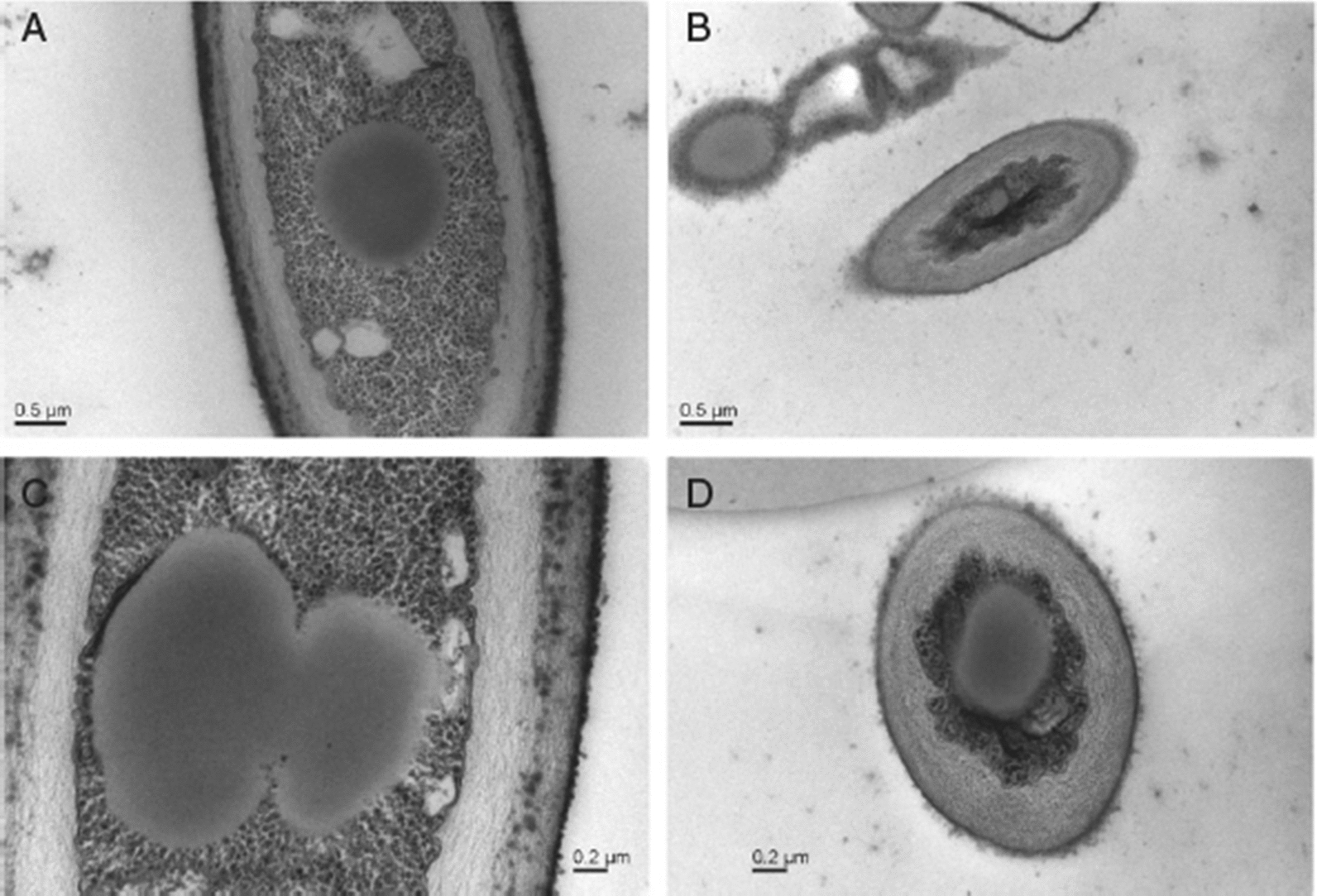
Transmission electron microscopy. **a**, **c** undamaged cell membrane and organized cell wall in cells with no RLs treatment, **b**, **d** smaller, deformed and indistinct cell wall in cells treated with RLs at 1000 µg/mL [68]. "(Reprinted (Journal of the Science of Food and Agriculture) with the permission from Wiley Online Library (License number 1066688-1)”

**Fig. 5:**
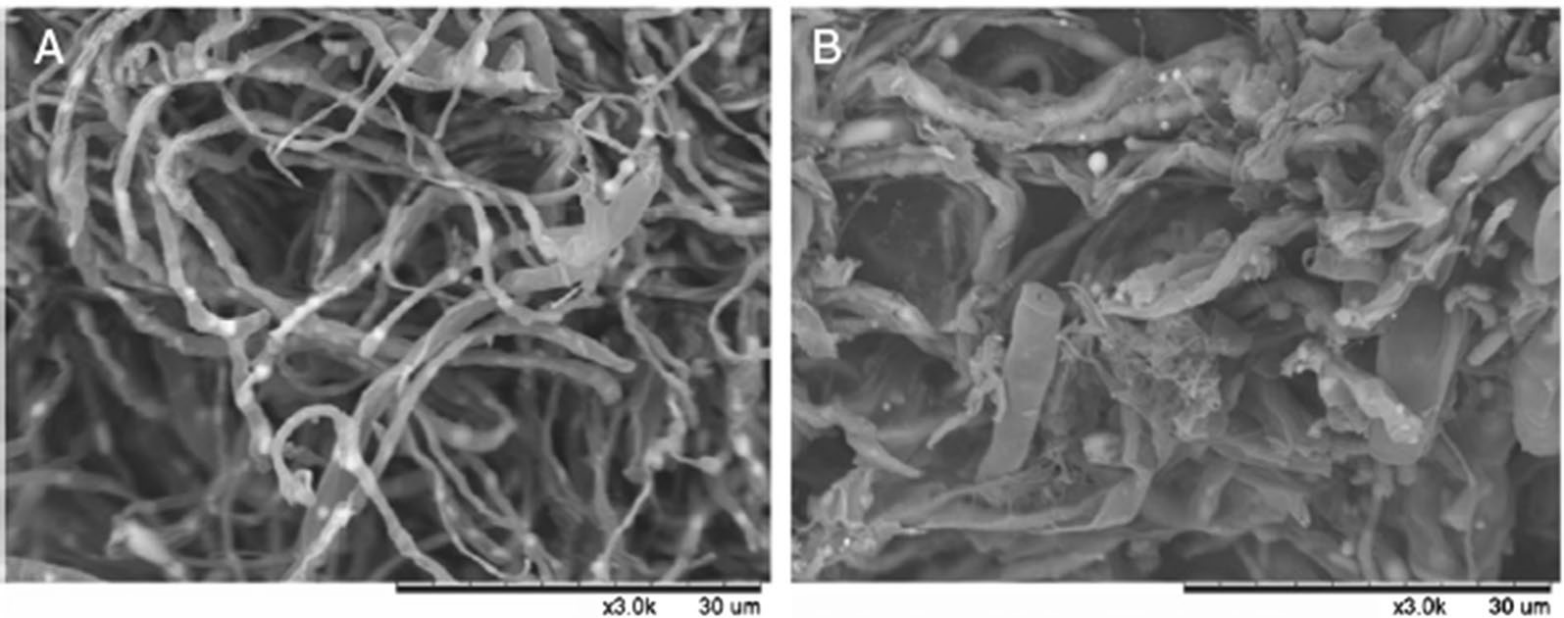
Scanning electron microscopy (SEM). **a** regular form of hyphae without RLs treatment, **b** shattered, irregular exteriors of hyphae when treated with RLs at 1000 µg/mL [68]. “(Reprinted (Journal of the Science of Food and Agriculture) with the permission from Wiley Online Library (License number 1066688-1)”

Yan et al., showed that rhamnolipids have evident antifungal effect against the post-harvest decay of *Lycopersicon esculentum* (cherry tomato) caused by *Alternaria alternata* [59]. Rhamnolipids can only inhibit the growth of fungus, so they required specific concentration to work efficiently, they inhibited the growth of fungus at 250 µg/mL, and above this concentration, they only hampered the growth of fungus but did not inhibit its growth. But if the concentration proceeded above 3000 µg/mL, rhamnolipids damaged the cells which diminished the cherry tomato’s potential to combat the fungal attack. Rhamnolipids alone did not exert any efficacious effect on fungal inhibition, but rhamnolipids along with laurel oil had a great inhibitory effect against *Alternaria alternata*. This might be due to the remodelling of the cell wall and plasma membrane of *Alternaria alternata* by rhamnolipids which permitted the laurel oil to invade inside the cytoplasm with ease to show their inhibitory effect. Rhamnolipids were reported to make changes in the cell membrane of *Alternaria alternata* (Fig. 4) and morphology of the hyphae (Fig. 5) resulting in the inhibition of mycelium growth and spore germination of the fungus which declined their growth. This study indicated that rhamnolipids can control the post-harvest damage of cherry tomato by preventing the mycelium growth and spore germination of *Alternaria alternata* [68].

#### Antifungal activity against dimorphic fungi (*Mucor circinelloides* and *Verticillium dahlia)*

Rhamnolipids are turned out to be effective against the two members of dimorphic fungi namely *Mucor circinelloides* and *Verticillium dahlia* isolated from the tomato plant [69]. Sha and Meng in their study showed that rhamnolipids had inhibitory effect on the filamentous and yeast-like form of both members of dimorphic fungi [69]. In *M. circinelloides*, rhamnolipids inhibited the mycelium growth by 59% at 200 µg/mL concentration on potato dextrose agar (PDA) plates. Further biomass accumulation and spore germination of *M. circinelloides* in yeast and mycelium growth were inhibited by rhamnolipids. Rhamnolipids inhibited 91% biomass accumulation and 95% spore germination of mycelium growth in shaking culture (aerobic) at 200 µg/mL concentration of rhamnolipids; inhibited 65% spore germination and 85% biomass accumulation of yeast-like form in static culture (microaerophilic). Rhamnolipids inhibited the colony growth of *V. dahlia* by 73% at 60 µg/mL, also lowered the accumulation of biomass by 60% in mycelium growth and 70% in yeast growth at 120 µg/mL and inhibited the spore germination by 50% in both growth forms [69].

#### **Antifungal activity against*Aspergillus niger*and*Aspergillus carbonarius***

**Fig. 6:**
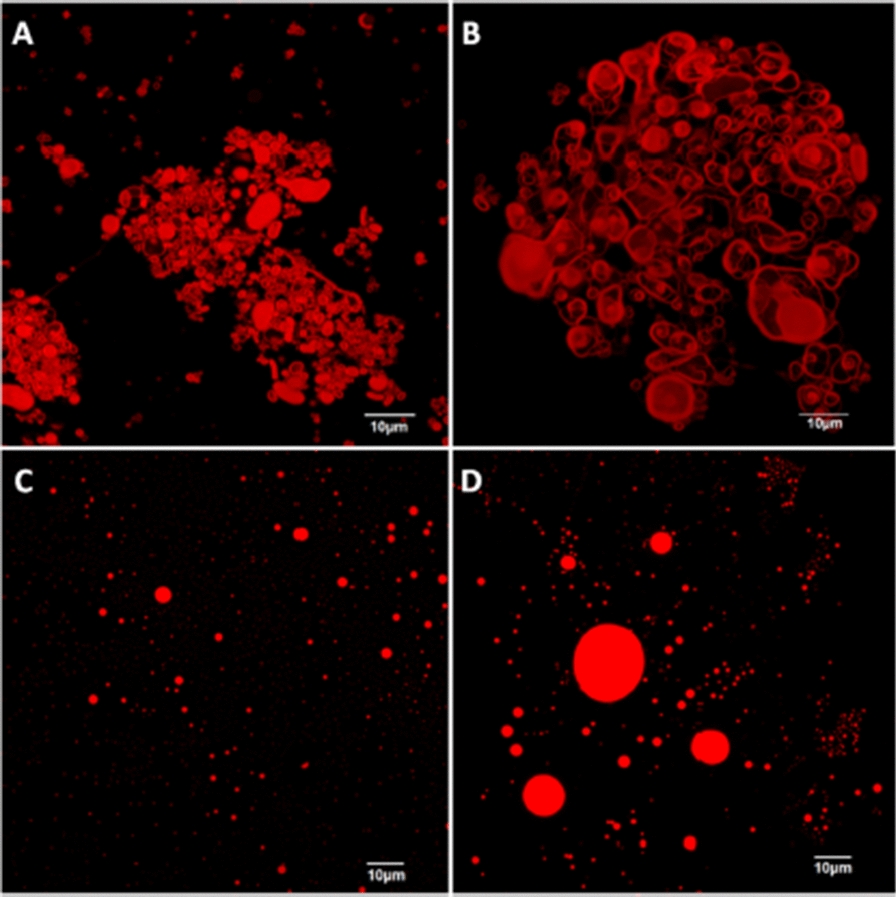
Confocal Scanning Laser Microscopy (CSLM). **a**, **b** giant vesicle-like structures developed in crude RLs, **c**, **d** spherical structures developed in mono- and di-rhamnolipids [61]

In a very important study, the growth inhibitory effects of cell-free supernatant obtained from *PA* strain 112 carrying rhamnolipids was checked against *Aspergillus* spp. The cell-free supernatant had 75.5% inhibitory effect against *Aspergillus niger* MUM 92.13 whereas exerted absolute inhibitory effect against *A. carbonarius* MUM 05.18. This might be due to the interaction of RL with the lipids present in the cell membrane resulting in the generation of ion channels and pores by interrupting the permeability and integrity of the cell membrane [70, 71]. Rodriguez and his coworkers also checked the antifungal activity of crude rhamnolipids against these fungi, but the inhibitory effect was less as compared to the cell-free supernatant [61]. Aggregation behavior of rhamnolipids was responsible for their biological activities. To further demonstrate this behavior of RLs, NaCl was added in the culture medium at 750–1000 mM concentration. A combination of crude RL mixture with different concentrations of NaCl showed complete inhibitory effect. As an example, against *A. niger* MUM 92.13 the inhibitory effects were observed at 875 mM of NaCl and against *A. carbonarius* MUM 05.18 at 375 mM of NaCl concentration. Di-rhamnolipids congeners also had an absolute inhibitory effect in the presence of NaCl, whereas mono-rhamnolipids had less inhibitory effect. The 875 mM concentration of NaCl resulted in reduced surface tension (31.7 ± 0.1 mN/m) but not cmc values in di-rhamnolipids whereas in mono-rhamnolipids cmc values were reduced (25 mg/L) but not surface tension. These results were further examined through Confocal Scanning Laser Microscopy (CSLM), in case of crude rhamnolipid mixtures, giant vesicle-like structures were formed although spherical structures were formed in mono and di-rhamnolipids (Fig. 6). Their results specified that NaCl increased the aggregation behavior of rhamnolipids ultimately resulting in their enhanced antifungal activity [61].

### Rhamnolipids in oxidative stress response

Rhamnolipids are also known to reduce the oxidative stress caused by *Alternaria alternata* by inducing resistance and defense mechanisms in cherry tomato [72]. Low levels of oxidative stress trigger defense mechanism in fruit against the fungal pathogen, however high levels result in the decay of fruits [73]. Yan et al., showed that within 12 h of treatment of cherry tomatoes with rhamnolipids and pathogen, the intracellular H_2_O_2_ levels increased but after 12 h of treatment there is a drastic decrease in the H_2_O_2_ levels [72]. So, there should be an equilibrium between ROS generation and degeneration in the initial phase to increase the resistance in cherry tomato. It was also noted that rhamnolipids increased the levels of catalase and superoxide dismutase, which quench the high ROS. It was also found that glutathione (GSH) also eliminated the intracellular ROS by the activation of glutathione reductase which converted the oxidized glutathione into glutathione. When the fruit was treated with RLs and pathogen, there was an increased content of GSH in the 12–36 h interval, but there was decreased content of both H_2_O_2_ and GSH when the fruit was treated with pathogen accompanied by RLs after 12 h, indicating that RLs activated H_2_O_2_ generation which led to the production of GSH to quench the high ROS [72]. Thus, RLs can induce resistance in cherry tomato against *A. alternata* infection through their response to oxidative stress by triggering the production of antioxidant enzymes to eliminate the excessive ROS.

### Rhamnolipids against foodborne pathogens

Foodborne bacterial pathogens like *Escherichia, Bacillus* spp., *Listeria, Campylobacter* spp., *Staphylococcus* spp, *Salmonella* spp. and *Clostridium *spp. cause various foodborne diseases (FBD) and WHO reported that these diseases result in deaths of about 4,20,000 people every year worldwide [45, 74–76]. Bacterial pathogens are developing resistance to chemical preservatives, therefore there is need of natural preservatives which could check the growth of pathogens without affecting the food quality [45]. Rhamnolipids can be used as an alternative to stop the contamination of food because of their antimicrobial property towards the wide spectrum of microbes as shown in Table 2 [13, 45, 77–79].

**Table 2:** List of microorganisms against which RLs show antimicrobial activity

Microorganisms	Species
Fungi	*Phytophthora *spp., *Campylobacter, Fusarium graminearum*, *Botrytis *spp.*, Phytophthora* *capsici* and *Mucor *spp.
Gram-positive bacteria	*Staphylococcus *spp.*, Clostridium perfringens, Bacillus subtilis, Listeria *spp.*, Bacillus *spp.
Gram-negative bacteria	*Escherichia *spp.*, Salmonella *spp., *Enterobacter aerogenes*

Magalhaes and Nitschke used 32 *Listeria monocytogenes* (food borne pathogen) cultures to test the antimicrobial activity of rhamnolipids [13]. They reported that 90.6% cultures showed sensitivity towards rhamnolipids (MIC 78-2500 mg/mL). Rhamnolipids were bacteriostatic in their activity since only 4 cultures had minimal bactericidal concentration (MBC). Rhamnolipids and nisin, which is antimicrobial peptide isolated from *Lactococcus lactis*, were investigated for their synergism owing to their common target, cytoplasmic membrane, of *Listeria*. The *L. monocytogenes* strain L12 was more sensitive in comparison with L17. Checkerboard test was carried out to examine the interaction between rhamnolipids and nisin, and the results indicated that fractional inhibitory concentration (FIC) index was less than 0.5 in both L12 (0.18) & L17 (0.078) isolates representing a great synergistic action. Because of the antimicrobial trait of RLs and their synergism with nisin, they can be used in food industry to control the *L. monocytogenes* borne diseases.

**Fig. 7:**
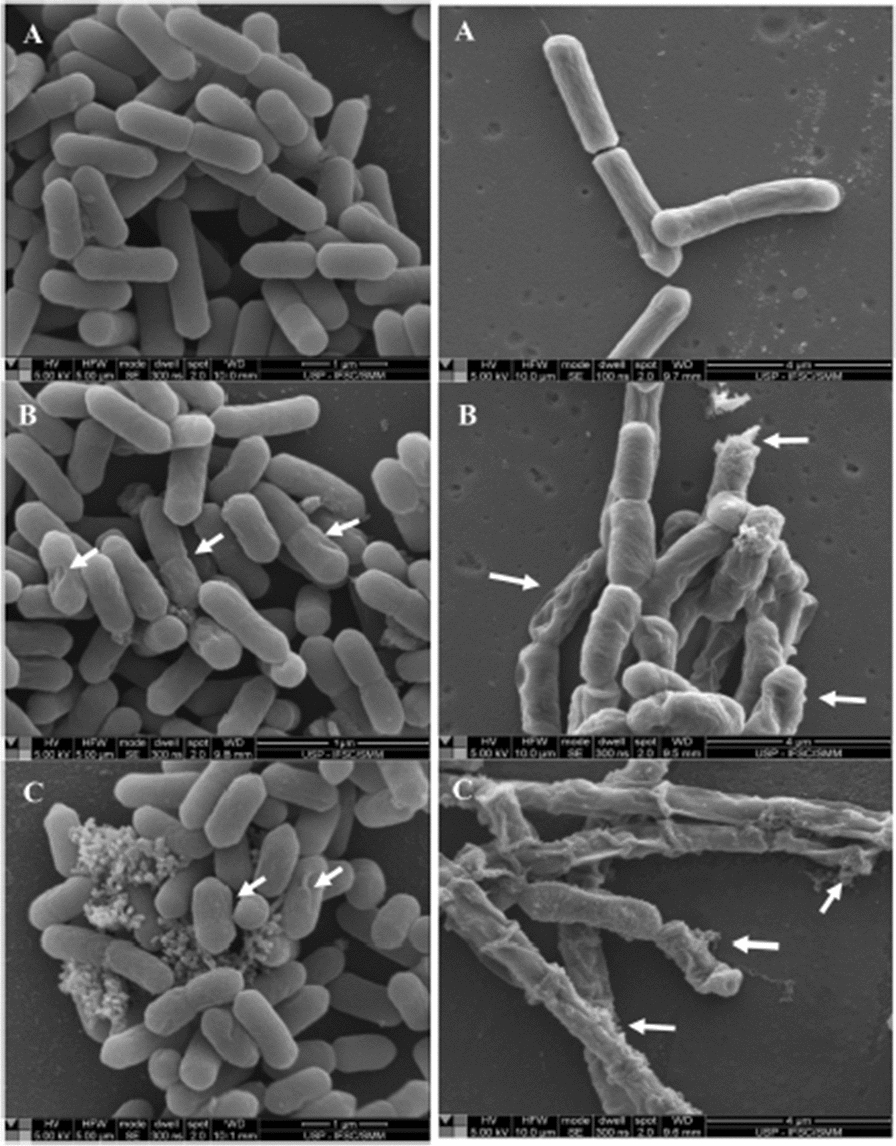
Scanning electron microscopy photographs presenting the results of ***Listeria monocytogenes*** (left), ***Bacillus cereus*** (right). **a** represents the untreated cells having regular shapes and structures, **b** represents RL-treated cells at MIC having distorted and irregular cells, **c** represents cell injury and out flow of intracellular material from RL-treated cells at ×100 MIC. Arrows specify the disruption of cells, enclosing of the cell membrane and outflow of intracellular material [45]. “(Reprinted (Food Research International) with the permission from Elsevier (License number 4,918,291,153,807)”

A study was conducted by de Freitas Ferreira et al., at varied pH values to check the antimicrobial activities of RLs against various foodborne bacterial pathogens because pH leads to the development of microbes on food [45]. At pH 7, RLs repressed the growth of *L. monocytogenes, Bacillus cereus* exhibiting MIC = 156.2 µg/mL & MIC = 19.5 µg/mL values (Fig. 7) but showed partial inhibitory effect on *Staphylococcus aureus.* However, at pH 5 and pH 6, 39.1 µg/mL concentration of rhamnolipid exhibited bactericidal and bacteriostatic activity against *Staphylococcus aureus.* In case of gram-negative bacteria *Escherichia coli* and *Salmonella enterica*, rhamnolipids had not shown any antimicrobial effect at different pH levels because of the protective behavior of outer cell membrane of gram-negative bacteria. By using the till-kill assay it had been found that RLs completely killed the *Bacillus cereus* cells after 30 min of treatment by destructing the cytoplasmic membrane and by lowering the surface hydrophobicity. Thus, rhamnolipids can control the growth of gram-positive bacteria in acidic food by enhancing their antimicrobial activity in acidic environment. We can conclude that rhamnolipids can be used in food industry to prevent the spoilage of food without affecting the quality of food.

### **Antibiofilm activity against*Staphylococcus aureus***

**Fig. 8:**
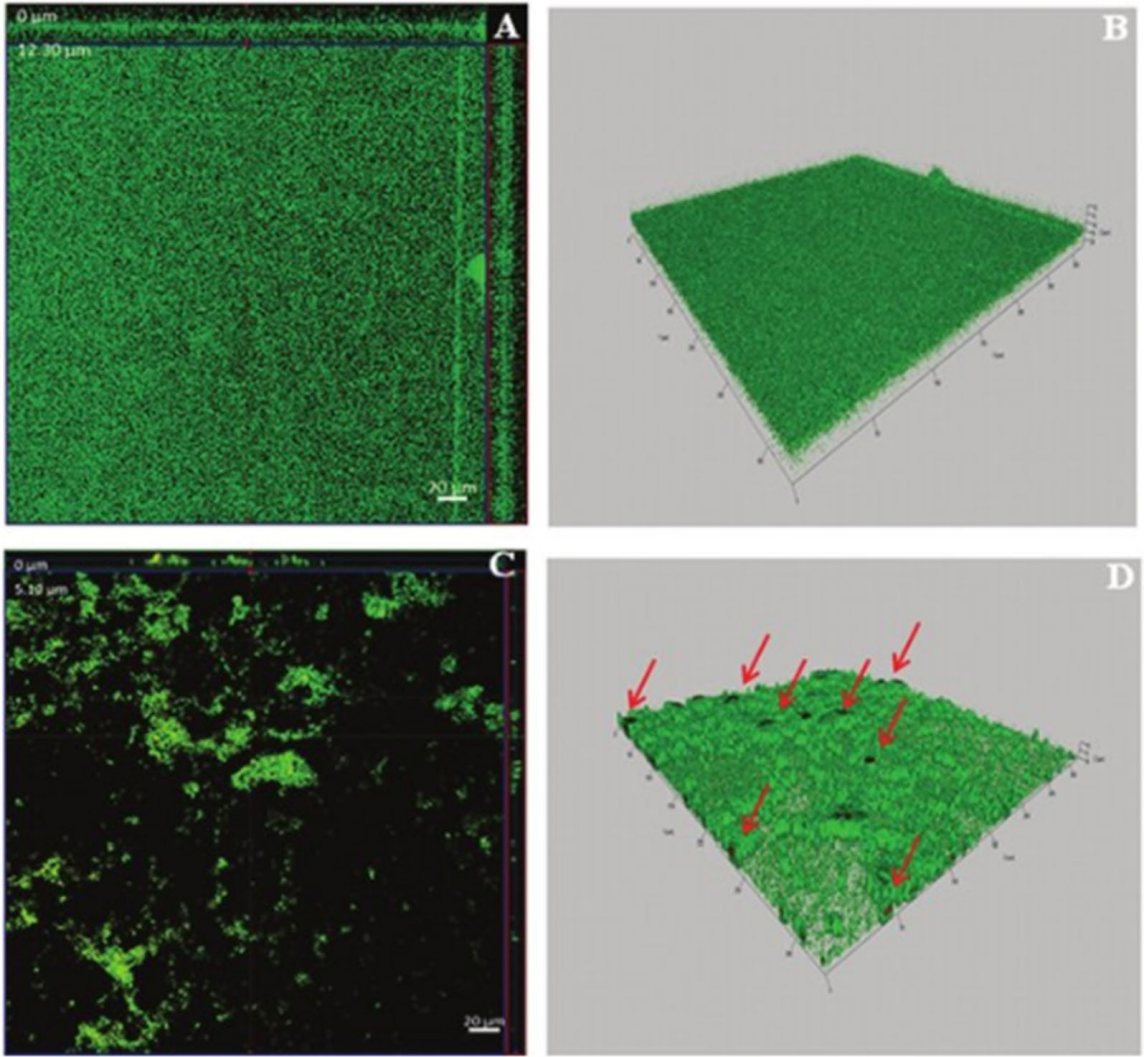
Confocal microscopy of biofilm formed in skim milk. **a**, **b** are orthogonal and 3D pictures of control biofilm, whereas **c**, **d** pictures show biofilm treatment with 0.05% of RL for 2 h at 4 °C. Control biofilm was condensed, 12 µm thick and showed the prevalence of living cells, while RL treated biofilm was 5 µm thick and showed disturbance in the matrix. Green cells are live cells and red cells are dead cells. Dead cells are represented by arrows [81]. "(Reprinted (Journal of Dairy Science) with the permission from Elsevier (License number 4,918,291,309,239)”

*S. aureus* has the potential to develop the biofilm in food like dairy, fish, poultry, ready-to-eat food and meat resulting in damage, contamination and decay of food [79–81]. Rhamnolipids act as antibiofilm agents to prevent the emergence of biofilm formation on food [82]. It has been shown that skim milk and nutrient broth were used as a culture media to examine the ability of rhamnolipids to remove the biofilm formed by *S. aureus* on polystyrene plates [81]. 0.1% concentration of RLs removed only 35% biofilm formed in nutrient media and 86.9% biofilm formed on skim milk at 25 °C for 2 h. Biofilm destruction results might be due to the decrease of capillary forces, contact angle, interfacial and surface tensions [83]. RLs were less effective to nutrient-biofilm in comparison with milk-biofilm; this is due to the presence of carbohydrates in milk which make the possible interaction between rhamnolipids and carbohydrates following the separation of biofilms (Fig. 8) [81]. These results suggest that rhamnolipids disrupt the emergence of biofilm on two basis (1) composition of biofilm matrix, (2) nutrient media, therefore suggesting their potential role in food industry especially in dairy.

### RLs modulating outer membrane protein (OMP)

OMP also known as integral membrane protein is synthesized by gram negative bacteria in the cytoplasm [84]. OMPs are β-barrel proteins which comprise of 8–22 β-strands [85]. OMPs carry out varied roles, for instance they work as enzymes (proteases, palmitoyl transferases and lipases), siderophore receptors, nutrient uptake channels and protein translocators [84, 86]. OMPs comprise of OmpA (heat-modifiable protein), porins (OmpC, OmpD, OmpE, OmpF) and the Braun’s lipoprotein [87]. In *PA*, 30% of the proteins present in extracellular matrix were identified as OMPs which were procured from outer membrane vesicles [88].

**Fig. 9:**
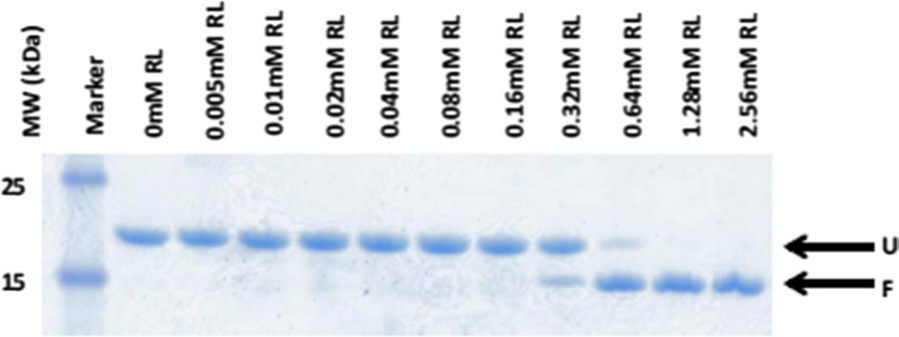
SDS-PAGE band shift assay for the confirmation of TM-OmpA folding. U is unfolded protein whereas F is folded protein [43]. “(Reprinted (FEBS Letters) with the permission from Elsevier (License number 4,918,300,176,808)”

OMPs are folded and stabilized by surfactants and phospholipid vesicles [89]. Only neutral and zwitterionic surfactants can fold the OMPs, but their concentration must be above the critical micelles concentration (cmc) [43]. Although anionic biosurfactant rhamnolipid can also fold OmpA above the cmc which was shown by Anderson et al., in their study [43]. As shown in band shift assay in SDS PAGE, the folding of TM-OmpA (transmembrane domain of OmpA) begin at 0.32 mM RL concentration which further increases at 0.64 mM RL concentration and above. At this concentration only folded rhamnolipids dominated. as shown in Fig. 9.

In proteolysis assay, trypsin was used to degrade the folded and unfolded proteins. Trypsin digested TM-OmpA protein present only in the buffer but obstructed the digestion of TM-OmpA protein which was surrounded by rhamnolipids and dodecyl maltoside (non-ionic surfactant) micelles at 10 mM concentration.

**Fig. 10:**
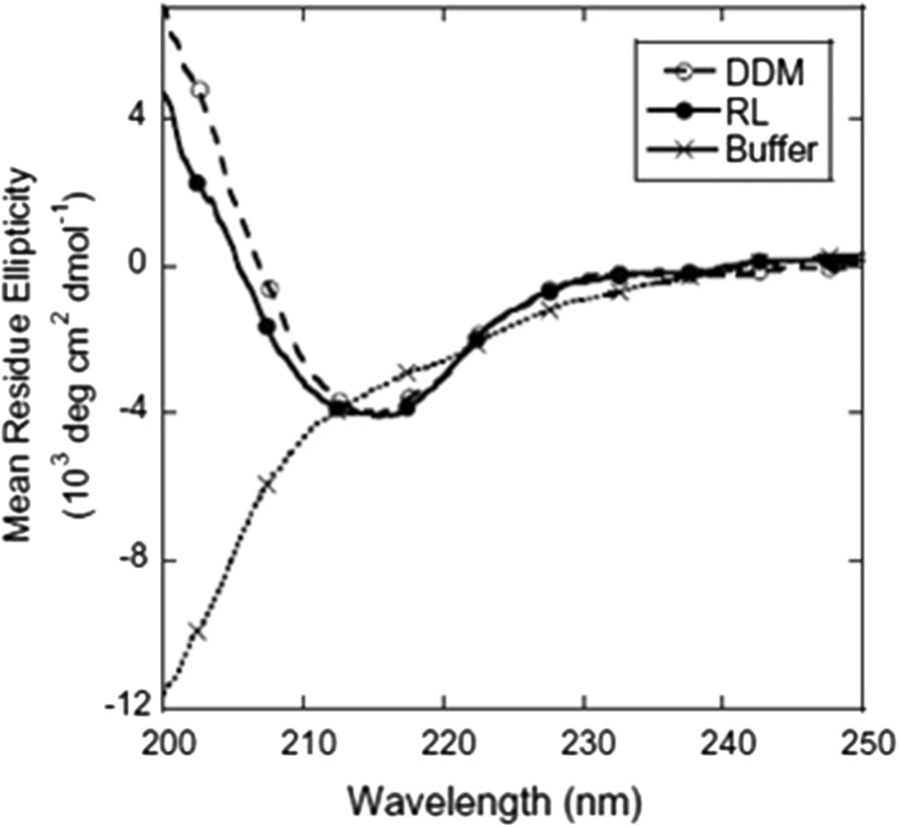
Far-UV Spectrum of TM-OmpA in buffer, RL and DDM [43]. "(Reprinted (FEBS Letters) with the permission from Elsevier (License number 4,918,300,176,808)”

In CD spectroscopy, Far-UV spectrum showed that at 214 nm, RL and dodecyl maltoside (DDM) had immense β-sheet structure, also identical in both RL and DDM, whereas in buffer, unstructured random coil was observed as shown in Fig. 10.

All these three assays confirmed that RL micelles fold the TM-OmpA. Futhermore, rhamnolipids decreased the thermostability of TM-OmpA compared to non-ionic surfactant DDM but increased in comparison to SDS [43]. But the native structure of OMPs unfold at very low rate because of their kinetic stability under severe circumstances, consequently the interaction of RL with OMPs is not affected by the decreased thermostability in comparison with DDM [90]. Therefore, OMPs can also be folded in anionic biosurfactant rhamnolipid above the cmc.

### Potential role of rhamnolipid nanoparticles for drug delivery

One of the main focus of current nano-research is on the synthesis of the nanoparticle by microorganism and this green chemistry approach interconnects the field of nanotechnology with microbial biotechnology. In order to develop eco-friendly approaches to synthesize the bioactive nanoparticles, biosurfactant has emerged as promising alternative, not only for the synthesis but also for the stabilization of nanoparticles. Among several biosurfactant, rhamnolipids, has been used in the synthesis of nanoparticles as a stabilizing agent by various groups [91–98].

Considering the potential of rhamnolipids, researchers started to investigate the role of these biosurfactant as nanoparticles for drug delivery. In that context one of the pioneer study which is conducted by Müller et al., used several rhamnolipids prepared either by chemical synthesis or commercially purchased, as nano-carriers for drug delivery to skin in ex vivo system [47]. They have used rhamnolipids nanoparticles loaded with different hydrophobic drugs like Nile red, dexamethasone, or tacrolimus for the skin delivery studies. Authors have clearly demonstrated that the loading of these rhamnolipids with hydrophobic drugs is achievable (up to 30% of drug loading). Further, by ex-vivo analysis it was demonstrated that fluorophore Nile reds loaded with rhamnolipids nanoparticles when applied to the isolated human skin, can efficiently deliver the Nile red into the skin and did not cause toxic effects at concentration higher than cmc values. This study opens a new dimension to use these rhamnolipids as an alternative drug delivery system.

**Fig. 11:**
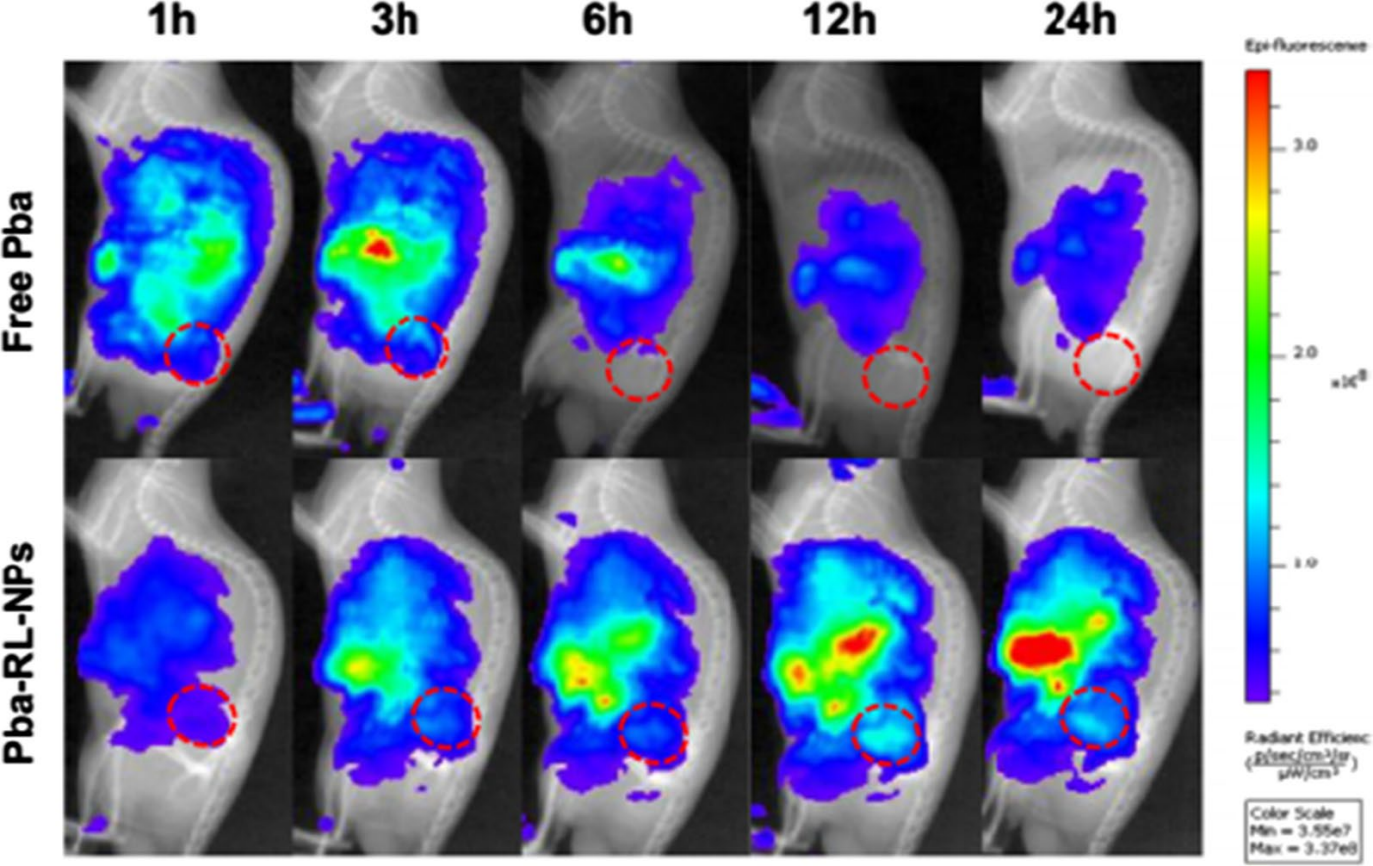
Real-time near-infrared fluorescence (NIRF) images of SCC7 tumor-bearing mice after intravenous injection of free Pba and Pba-RL-NPs [99]. "(Reprinted (Nanomedicine: Nanotechnology, Biology, and Medicine) with the permission from Elsevier (License number 4,918,300,392,622)”

**Fig. 12:**
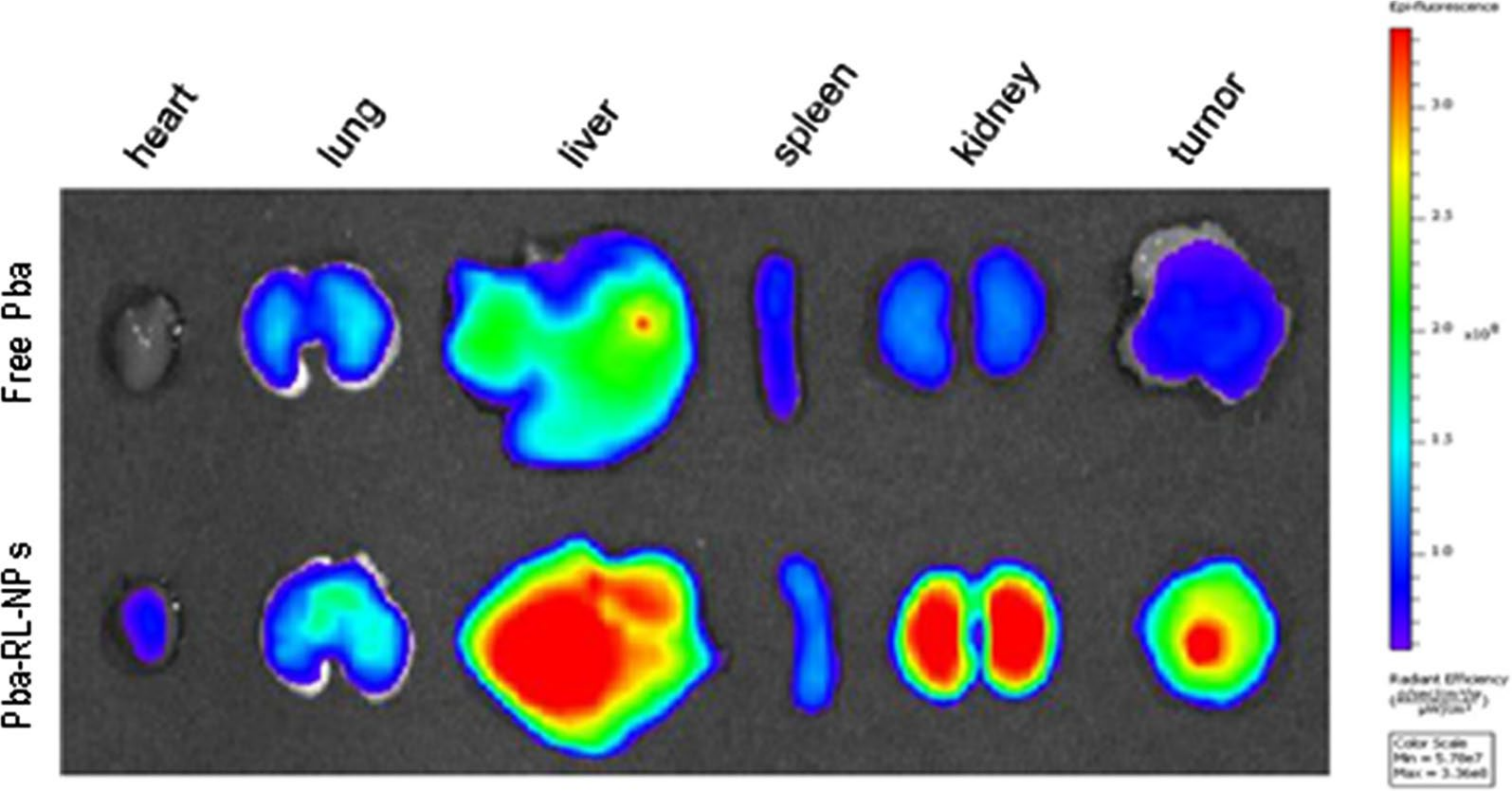
Ex vivo NIRF images of the dissected tumors and major organs of SCC7 tumor-bearingmice 24 h post-injection of free Pba and Pba-RL-NPs [99]. “(Reprinted (Nanomedicine: Nanotechnology, Biology, and Medicine) with the permission from Elsevier (License number 4,918,300,392,622)”

In 2019, an elegant study used rhamnolipids nanoparticles loaded with hydrophobic photosensitizer, “pheophorbide a” and injected it intravenously into the SCC7 tumor bearing mice. Interestingly, not only there was significant high level of accumulation of “pheophorbide a” carrying nanoparticles, Pba-RLNP in tumor tissue (Fig. 11) but the tumor was also suppressed by photodynamic-therapy (Fig. 12) [47].

Of the scope of the potential applications reviewed here, it is anticipated that rhamnolipid nanoparticles might enhance the cancer chemotherapy through their antitcancer activities and enhanced permeability and retention effects leading to the agglomeration of drugs inside the cancer cells. Emergence of biofilm is a major concern in food industry because they have some adverse effects like corrosion of appliances, blockage of pipelines, contamination and decay of food. They have severe impacts on the heath of cosumers as well as affecting the economy of the process. Rhamnolipids act as antibiofilm and antiadhesive agents to destruct the formation of biofilm by disturbing the solidarity of the cell membranes through their insertion into the lipid bilayer. Thus, rhamnolipids can be used farther as biofilm controlling strategies to prevent the decay of food. Their antifungal properties make them suitable to prevent the post-harvest decay of fruits and vegetables.

## Conclusion

In today’s scenario, rhamnolipids (surface active compounds) are very important due to their non-toxic nature, high biodegradability, low surface tension and cmc values, high emulsifying activity and having properties like antimicrobial, antitumor, antibiofilm, antifungal. These diverse properties of rhamnolipids make them effective to be used as the alternative to chemical surfactants and additives in various industries to overcome the hazardous effects of fungus, microbes and hydrocarbons on microorganisms, plants, animals, and humans. Rhamnolipids exhibit cytotoxicity against leukemic, cervical, breast and bladder cancer cells, disrupt the biofilm formation in dairy, eliminate the oxidative stress by activating antioxidant enzymes (SOD, CAT and GR), and fold the outer membrane protein OmpA. Rhamnolipids also prevent the contamination and post-harvest decay of food as well as improve the quality of food by executing morphological changes on the cell membrane of fungi and bacteria. MIC values of rhamnolipids show that they are used in low concentrations which make them as potent antimicrobial, antitumor and antifungal agents. Rhamnolipids possess varied physiochemical properties and have biological importance which make them potent for their future uses in cosmetic, pharmaceutical, food and health-care industries. Immunomodulatory activities of rhamnolipids make them influencial to secrete the pro-inflammatory cytokines through the activation of immune cells. RLs are also used as an alternative for the synthesis and stabilization of nanoparticles to be used in drug delivery system. In future, they can be used in green technologies as promising agents as they are produced from safe microorganisms and naturally occurring sustainable resources, active at low level and with little impact on the environment.

## Data Availability

Not applicable.

## Abbreviations

cmc: Critical micelle concentration
RL: Rhamnolipids
HAA: Primarily β-Hydroxydecanoyl-β-Hydroxydecanoate
ACP: Acyl carrier protein
CoA: Coenzyme A
MIC: Minimum inhibitory concentration
PDA: Potato dextrose agar
CSLM: Confocal scanning laser microscopy
ROS: Reactive oxygen species
H_2_O_2_: Hydrogen peroxide
SOD: Superoxide dismutase
CAT: Catalase
GSH: Glutathione
GR: Glutathione reductase
FBD: Foodborne diseases
MBC: Minimal bactericidal concentration
FIC: Fractional inhibitory concentration
OMPs: Outer membrane proteins
DDM: Dodecyl maltoside
12-HETE: 12-Hydroxyeicosatetraenoic acid
hBD-2: Human beta defensin-2
DAG: Diacylglycerol
Pba: Pheophorbide a
Pba-RLNP: Pheophorbide a-loaded rhamnolipid nanoparticles
NIRF: Near-infrared fluorescence
SEM: Scanning electron microscopy
